# Flood risk assessments at different spatial scales

**DOI:** 10.1007/s11027-015-9654-z

**Published:** 2015-05-22

**Authors:** H. de Moel, B. Jongman, H. Kreibich, B. Merz, E. Penning-Rowsell, P. J. Ward

**Affiliations:** 1 Institute for Environmental Studies, VU University, Amsterdam, Netherlands; 2 GFZ German Research Centre for Geosciences, Helmholtz Centre Potsdam, Potsdam, Germany; 3 Flood Hazard Research Centre, Middlesex University, London, UK

**Keywords:** Flood risk, Adaptation, Risk assessment, Spatial scales

## Abstract

Managing flood risk, i.e. both the hazard and the potential consequences, is an important aspect of adapting to global change and has gained much traction in recent decades. As a result, a priori flood risk assessments have become an important part of flood management practices. Many methodologies have been set up, ranging from global risk assessments for the world as a whole, to local assessments for a particular stretch of a river/coast or small town. Most assessment frameworks generally follow a similar approach, but there are also notable differences between assessments at different spatial scales. This review article examines these differences, for instance those related to the methodology, use of assessments and uncertainties. From this review, future research needs are identified in order to improve flood risk assessments at different scales. At global/continental scale, there is a clear need for harmonised information on flood defences to improve assessments. Furthermore, inclusions of indirect economic effects at the macro-/meso-scale would give a better indication of the total effects of catastrophic flooding. At the meso-/micro-scale, there is an urgent need to improve our understanding of the effects of flooding on critical infrastructures, given their importance to society, the economy, emergency management and reconstruction. An overarching theme at all scales is the validation of flood risk assessments, which is often limited. More detailed post-disaster information would allow for improved calibration, validation and thus performance of flood risk models. Lastly, the link between spatial scales also deserves attention, for instance up- or downscaling methodologies.

## Introduction

Time and again, floods around the world illustrate the devastating impact they can have on societies. In October 2012 super storm Sandy wreaked havoc in New York and New Jersey, United States of America (USA) causing roughly US$60 billion damage (Aerts et al. 2013a, b), and in June 2013 central Europe saw floods in the Elbe and Danube basins, causing a loss of approximately €12 billion and dozens of fatalities (Schröter et al. 2015; Munich 2013). Managing this risk is important from both a societal and economic perspective in order to reduce damages and losses and to minimise or avoid human suffering. With risk being a combination of the hazard and its potential consequences, flood risk can thus be managed by reducing the probability or magnitude of a flood (the hazard) or by reducing the consequences it may cause. In the last couple of decades, the concept of managing flood risk has gained much traction (Hooijer et al. 2004; Petrow et al. 2006; Van Alphen and Van Beek 2006; IPCC 2012; UNISDR 2013), which is exemplified by the European Floods Directive, which was adopted in 2007 (2007/60/EC). This approach of managing risk takes a more holistic view, by explicitly covering all aspects (e.g. prevention, mitigation, preparation, response, recovery) of the disaster management cycle (Lumbruso 2007; De Moel and Aerts 2008; Kreibich et al. 2014), instead of focussing mainly on flood prevention. As such, flood risk management is becoming an important process for adapting to a constantly changing environment due to, for instance, climate change, population growth and economic change.

Adequate management of floods is reliant on a priori assessments of flood events and their consequences. Such assessments give insights into what can be expected, and thereby open up the discussion on how to tackle such situations. Moreover, such assessment frameworks can be used to evaluate measures in a standardised way, support decision-making on possible measures that can be taken and prioritise areas where action is required. Over the past couple of decades, a large amount of research has focused on developing such flood assessments at various spatial scales, for a variety of purposes. Whilst the central approach is quite general between different assessments, there are notable differences in terms of methodology and the use of those assessments. Such differences are the result of many factors, including data availability and the applicability of methods at different scales. However, with increasing computational capacity and a wider availability of detailed data, opportunities are arising to enable the scaling up of detailed methods to larger scales or to start using global methods for more local purposes in data scarce regions. For this, it is necessary to understand the different methodologies, limitations and uses of flood risk assessments at different spatial scales.

This review paper aims to provide an overview of the current state and development of quantitative flood risk assessments at different scales and to facilitate learning from assessments at different scales. In the remainder of this review, we will first provide a general framework of flood risk assessments and then address the methods, limitations and uses of assessments at four different scales (supra-national, macro, meso, micro). An overview of assessment characteristics at different scales will then be discussed before providing the main lessons learned and identifying future research needs for different scales and for flood risk assessments generally.

## Flood risk assessments

Assessing flood risk is an interdisciplinary task, combining various sources and types of information and models. Such assessments attempt to estimate, a priori, what possible flood events may look like (i.e. flood extent and inundation depth), how probable they are and what are the possible consequences of such a flood may be. In engineering and natural sciences, the conceptual framework followed is that risk is a function of hazard, exposure and vulnerability (Kron 2002; UNISDR 2013), though concepts are sometimes differently interpreted by different researchers (Klijn et al. 2015).

**Fig. 1:**
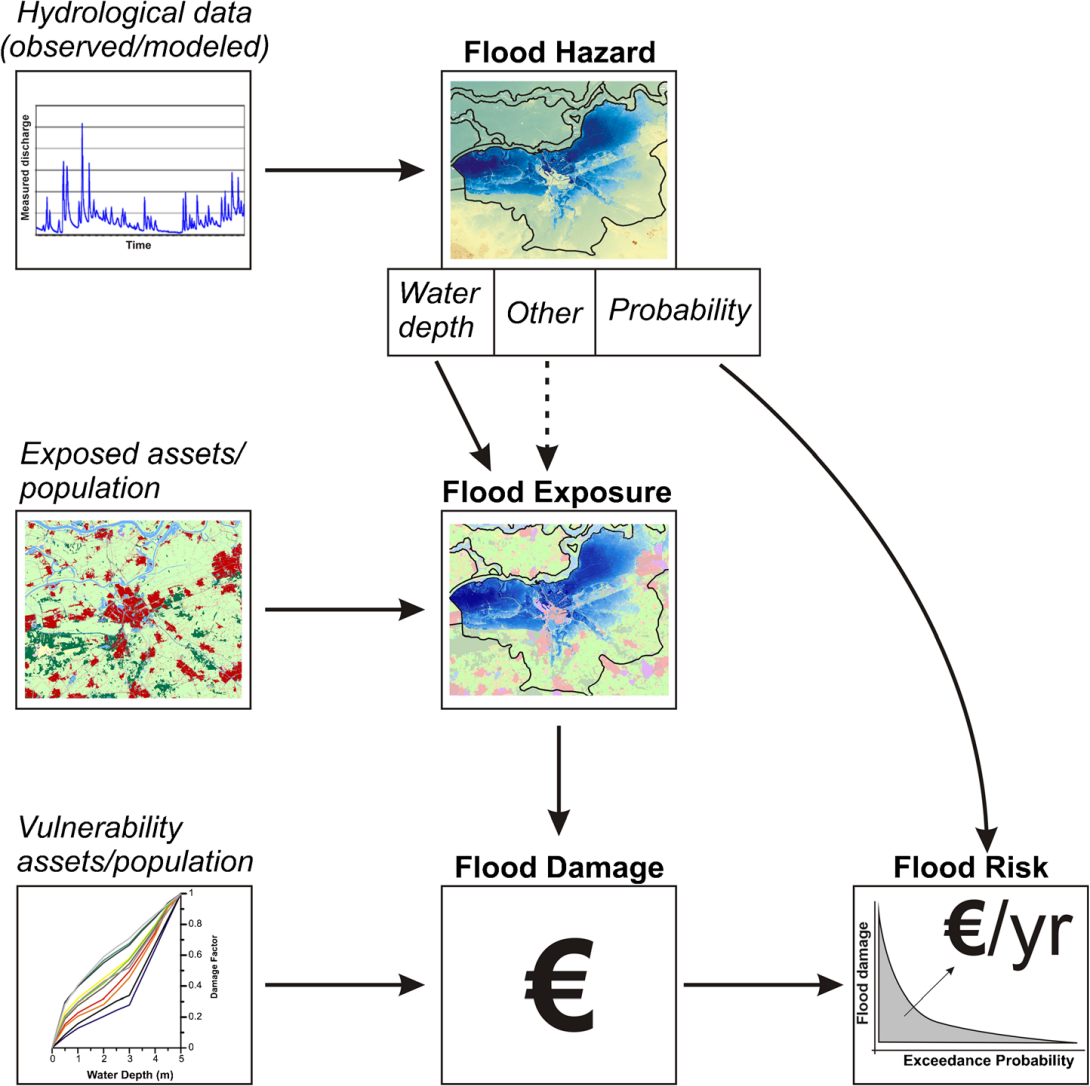
Conceptual overview of general flood assessment (Merz and Thieken 2004)

Flood risk assessments start with an assessment of the flood hazard, which indicates the probability and intensity of a possible event (hazard assessment; e.g. Pappenberger et al. 2012; Alfieri et al. 2013). This hazard information can be overlaid with socio-economic information, such as land use data, building datasets, information on population, regional gross domestic product (GDP), etc. Doing so will give an indication of what is actually exposed to flooding (i.e. exposure assessment; e.g. Jongman et al. 2012a). Information on the exposure can be combined with information on the vulnerability of such assets (for instance through damage curves) and the hazard characteristics to estimate the potential damage (damage assessment; e.g. Kreibich and Thieken 2008; De Moel and Aerts 2011). A generalised procedure to estimate direct monetary damage consists of the following: (1) Classification of elements at risk by pooling them into homogeneous classes. (2) Exposure analysis and asset assessment by describing the number and type of elements at risk and by estimating their asset value. (3) Susceptibility (or vulnerability) analysis by relating the relative damage of the elements at risk to the flood impact (Merz et al. 2010a, b). When total damages are calculated for several events with different probabilities, an expected annual damage can be calculated, usually referred to as risk (risk assessment; e.g. Aerts et al. 2013a, b; Falter et al. 2015a) (Fig. 1). Note that there are various other factors that influence flood risk, which may or may not be included in flood risk assessment as they are sometimes difficult to include in a quantitative analysis. Such factors include warning time, preparedness, social and economic resistance, health, etc.

### Hazard

Generally, flood assessments start with assessing the possible danger of the physical flooding itself or flood hazard. There are many dimensions (duration, velocity, debris, water depth, etc.) to the flood hazard, which makes this far from straightforward. Moreover, the methodology for such an assessment depends very much on the scale and specific characteristics, such as the geographic setting and the presence of defensive structures, the purpose of the assessment and the available data. The basis of each hazard assessment is, however, adequate observational data. Such observational data can be based on recorded flood events (i.e. derived from satellite imagery—Schumann et al. 2009; Schumann and Di Baldassarre 2010) but often entails time series of precipitation, river discharge, water levels at the coast, etc. Such time series, sometimes extended using stochastic weather generators (e.g. Te Linde et al. 2010a; Falter et al. 2015a; Ailliot et al. 2015), are subsequently used to derive probabilities and corresponding magnitudes of possible events. This can be done directly using frequency analyses to derive return periods for certain water levels (e.g. D’Onofrio et al. 1999) or discharges (e.g. Cunnane 1988). However, observational data, or data derived from observations (e.g. interpolated maps such as Adam and Lettenmaier 2003; Harris et al. 2013), can also be used as inputs in simulation models (hydrological and hydraulic models) which represent the flood generation processes in catchments and river systems. A typical example is the use of observed rainfall data as input for a hydrological catchment model. Such approaches have the advantage that hazard parameters can be estimated in space more easily, although there is also a variety of regional flood frequency analysis approaches with the aim of estimating flood hazard at ungauged locations based directly on observed flood data (Hosking and Wallis 1997). Much more information is required in the simulation model approach though, most importantly detailed topographic, but also land cover and soil characteristics. Besides allowing for more complete spatial coverage, using models also allows one to estimate various dimensions of the flood hazard that can be important for the potential consequences and decision-making, such as water depth (e.g. for determining damage to buildings), flow velocity (e.g. for determining damage to bridges; e.g. Kreibich et al. 2009), the rate of rise (e.g. for estimating fatalities), the flood volume (e.g. for designing flood retention measures) and the duration of inundation (e.g. for determining damage to agricultural crops; e.g. Tapia-Silva et al. 2011).

### Consequences

After the hazard assessment has been carried out, the hazard data (for example, a map of inundation extent and depth) can be combined with information on the exposure (i.e. the people, property, systems or other elements assets present in the hazard zones that can be subjected to potential losses; UNISDR 2013). This can take various forms, but often datasets on population, land use, buildings, etc. are used in this respect (Wünsch et al. 2009), or remote sensing data is utilised (Gerl et al. 2014). Such exposure assessments can be binary (i.e. affected or not affected the flood—Jongman et al. 2012a), or can involve various gradations, for instance, by taking various water depths into account (De Moel et al. 2011). Such exposure estimates usually relate to physical assets and people. However, also other exposure information can be incorporated, such as cultural values (using datasets on monuments and/or cultural heritage sites), parameters associated with indirect effects (using for instance exposure of infrastructure networks or GDP production) or long-term changes in exposure (related to population growth and economic development; e.g. Elmer et al. 2012; Tapia-Silva et al. 2011).

Many flood assessments estimate the direct consequences in monetary terms (Merz et al. 2010a). By doing so, different types of consequences are expressed in a single figure (e.g. euros/dollars/£), which allows for easy comparisons, for instance, between areas, over time, with other hazards or comparative evaluations of the effect of different measures (in terms of how much damage is reduced). Many flood damage assessments rely on water depth as main indicator of the hazard, though sometimes distinctions are made based on, for instance, the duration of flood (e.g. the ‘Multi-Coloured Manual’ in the United Kingdom (UK) Penning-Rowsell et al. 2013) or the flow velocity (e.g. HAZUS in the USA Scawthorn et al. 2006). This is usually done by differentiating different damage curves or combining different hazard characteristics into a new indicator (e.g. estimating the stability of buildings using velocity-stage-damage curves; Middelmann-Fernandes 2010). This is different, however, for damage to agricultural crops, for which the time of flooding (i.e. season) and the duration are decisive as opposed to water depth (Tapia-Silva et al. 2011).

The most commonly used approach to assess direct damages is based on depth-damage curves (Smith 1994; Merz et al. 2007). Such curves denote the vulnerability to flooding by relating the water depth to the damage this will cause to a specific type of asset, economic sector or land use category (see e.g. Merz et al. 2004; Freni et al. 2010; De Moel and Aerts 2011). However, not all approaches are based on damage curves. For example, Booysen et al. (1999) used questionnaires to estimate damages to industries in a case study area. Such an approach is particularly useful for categories that are very heterogeneous and need specific details (such as industrial land use). In addition, some multi-parameter models have been developed including: a conceptual model in the UK (Nicholas et al. 2001), a multi-variate regression model to estimate losses in private households in Japan (Zhai et al. 2005) and rule based models for loss estimation to companies and private households in Germany (Kreibich et al. 2010; Elmer et al. 2010). Such multi-parameter methods have the advantage that additional important factors that are often difficult to predict (e.g. warning time, contamination and resilience) can sometimes still be included in the assessment.

The development of probabilistic damage models seems to be an innovative enhancement, since they inherently provide quantitative information about model uncertainty. Probabilistic models using bagging decision trees or Bayesian networks have been successfully validated (Vogel et al. 2013; Merz et al. 2013; Schröter et al. 2014). These approaches have the advantage that more variables (than just inundation depth) can be included in the damage assessment and have shown the importance of factors like contamination, precaution and duration of the inundation (Thieken et al. 2005; Merz et al. 2013).

### Risk

When several damage assessments are performed for events with different probabilities, the results can be combined to derive an estimate of the monetary risk per year, also known as expected annual damage (EAD) or (average) annual loss (AAL). This is done by integrating the damage estimates over their probability of occurrence and corresponds graphically to the area underneath an exceedance-probability loss (EPL) curve (Meyer et al. 2009; Ward et al. 2011). Note that the probability used to integrate (i.e. the probability axis of an EPL curve) has to be associated with the probability of a certain amount of damage occurring, which is not necessarily the same as the probability associated with certain hydraulic conditions such as water levels (see e.g. Aerts et al. 2013a, b). Often the hazard probability is directly used though.

Such risk assessments thus combine the consequences of flood events of different magnitudes. They provide input for various follow-up activities, most notably cost-benefit analyses of measures (e.g. Saint-Geours et al. 2013) but also setting insurance schemes and premiums (e.g. Aerts and Botzen 2011). Whilst damage per year (which equals the integral under the EPL; Fig. 1) is a rather objective indicator for risk, it should be noted that it may not be appropriate for all circumstance as two situations with the same damage per year are not necessarily the same (Merz et al. 2009) because the shape of the risk curve can be different and is also an important characteristic of risk (see e.g. Bouwer 2010; Foudi et al. 2015). For instance, a situation with high probabilities, but low damage (no levees, few or adapted buildings) and a situation with low probabilities but high damage (many buildings protected by levees) could have the same expected annual damage. However, such situations are still perceived differently by various actors (insurers, society; e.g. Seifert et al. 2013). The first situation relates to relatively small events occurring frequently, which are easier to handle by emergency services and to cover by insurance, whilst the second situation entails very rare, but more catastrophic, events that could be very difficult to control and would instantly be a huge financial burden.

### Types of consequences

When assessing potential consequences of flooding, it is of course of key importance which consequences are considered and which ones are not (Meyer et al. 2013; Kreibich et al. 2014). Generally, consequences of natural disasters are differentiated into four groups, based on two criteria: direct vs. indirect consequences and tangible vs. intangible consequences (e.g. Messner et al. 2007; Jonkman et al. 2008). Direct consequences relate directly to the area and time of the flood event, whilst indirect consequences are often associated with consequences occurring outside the flooded area, or after the flood event. Tangible and intangible consequences refer to effects that can be monetized (tangible) and effects that are much more elusive to quantify or even monetize.

Recently, it has been recognised that catastrophic floods can have substantial effects on the economy, also outside the area affected and after the flood event (Okuyama and Santos 2014). Estimating these economic losses is far from straightforward, as even fewer observed data are available than for direct losses. Flood losses in one area or sector will affect other areas and sectors. Therefore, economic models are usually used, such as input-output or general equilibrium models (Rose and Liao 2005; Steenge and Bockarjova 2007; Hallegatte 2008; Li et al. 2013). Alternatively, a certain percentage of the direct damages can be taken as an indication of indirect losses, often based on the relevant modelling studies. Such estimates typically range from 30 up to 100 % of the direct damages and is generally thought to increase with the severity of the flood event (Hallegatte 2008; Koks et al. 2014a, b). Some empirical evidence on indirect losses is provided by Toyoda (2008), who estimated the indirect losses of the great earthquake in Kobe in 1995 at roughly the same size (i.e. 100 %) as the direct losses. Such indirect economic losses are very case and site specific though.

### Uncertainty

As with any quantitative modelling assessment, flood risk assessments are surrounded by uncertainties (Merz and Thieken 2005; 2009). The nature of this uncertainty can result from different types of uncertainty. Broadly speaking, this can be aleatory, epistemic or ontological uncertainty. Aleatory uncertainty relates to natural variation in the system (such as variation in rainfall over time, or spatial variation in roughness characteristics) and can in principle not be reduced. Epistemic uncertainty relates to inadequate knowledge of the system, resulting in the analyst having to make assumptions on how the system works. This uncertainty can, theoretically, be reduced with increased knowledge of the system, although this is in practice sometimes not easy. Lastly, ontological uncertainty refers to factors missing out of the analysis, i.e. incompleteness. This could be considered a subset of epistemic uncertainty, but it is much more difficult to include in formal uncertainty analyses.

## Assessments at different scales

Different scales call for different methods when it comes to assessing flood risks. In addition, assessments at different scales have different uses. In this section, we will touch upon these different scales. Drawing upon examples from international literature, the characteristics of the flood hazard assessment will be described, as well as the way consequences are assessed, which uncertainties are important and for what the assessments at different scales are used for.

We distinguish the supra-national, macro-, meso- and micro-scale. Obviously, the distinction between these scales is subjective. The supra-national scale concerns assessments of the entire globe or continent, encompassing a plethora of countries and river basins. The macro-scale refers to assessments of entire countries, for which usually consistent (national) data are present, but consists of many watersheds. The meso-scale is generally sub-national, referring to a certain province, watershed or large city. The micro-scale is the smallest scale considered, which relates to a town or specific river stretch.

### Supra-national (global, continental) scale

#### Importance and use

Over the last decade, there has been an increased demand from diverse stakeholders for flood risk assessments at continental to global scales. For instance, the United Nations International Strategy for Disaster Risk Reduction (UNISDR) now coordinates the production of the two-yearly Global Assessment Report (GAR) on Disaster Risk Reduction (UNISDR 2009, 2011, 2013). Continental flood risk assessments are important in Europe to support climate change adaptation policies (Van Renssen 2013) and develop robust public disaster relief funds (Hochrainer et al. 2010). As disaster risk modelling capabilities improve continuously, applicability and interest in global scale flood risk assessment is also increasing. For example, international financing institutes need strategic assessments of flood risk at the global scale for developing risk profiles and deciding where to invest in risk reduction activities, the (re-) insurance industry requires global scale assessments to assess their current and future risk portfolios, multi-national companies are interested in global scale risk assessments to identify possible risks in their supply chains and intra-national institutes can use the data for monitoring progress in risk reduction activities, such as those related to the implementation of the Hyogo Framework for Action (UNISDR 2007).

#### Method

An increasing number of scientific efforts have contributed to the understanding of trends in flood risk and the modelling of current and future risk at continental and global scales. Several studies (e.g. Barredo 2009; Bouwer 2011; Neumayer and Barthel 2011) have directly analysed past trends in flood losses. Generally, these studies have found that observed disaster losses have increased over the last half-century, with changes in exposure (e.g. wealth and population growth) being the main driver (IPCC 2012; Merz et al. 2012; Kundzewicz et al. 2013). However, Gall et al. (2011) found evidence of non-exposure driven increases in disaster losses (of various natural hazards) in the USA over the period 1960–2009, pointing to changes in hazard frequency/intensity as possible drivers of risk.

In recent years, several models have been developed to assess flood hazards, potential damages and risk at large scales. Methods have been developed and applied to assess flood hazard (Pappenberger et al. 2012; Rojas et al. 2012), exposure (Jongman et al. 2012a) and/or flood risk (Dilley et al. 2005; Hirabayashi et al. 2013; Feyen et al. 2012; Ward et al. 2013, 2014; Winsemius et al. 2013; Arnell and Lloyd-Hughes 2014) at continental and global scales. Some of these have been combined with projections of changes in hazard and/or exposure to assess the potential change in flood risk (or flood exposure) in the future (e.g. Jongman et al. 2012a; Hirabayashi et al. 2013; Rojas et al. 2013; Arnell and Lloyd-Hughes 2014; Jongman et al. 2014). The risk of coastal flooding has been assessed similarly on a global scale, by modelling storm surges heights, determining potential inundation areas using elevation data and using macro-economic and population data to determine the exposure in major coastal cities (e.g. Nicholls et al. 2008; Hallegatte et al. 2013). The results of the latter studies show that the increase in exposure to coastal flooding alone could lead to an eightfold increase in losses, with climate change potentially adding further to the increase in risk.

#### Uncertainty and validation

The accurate representation of vulnerability and flood protection has been the largest obstacle in large-scale flood risk assessment (Jongman et al. 2012a; Feyen et al. 2012). First efforts to include flood protection measures in risk assessment have been presented on European (Jongman et al. 2014) and global (Hallegatte et al. 2013, Ward et al. 2013) scales. These studies show that the flood protection standards assumed in the modelling process have a large effect on the computed risk estimates, which illustrates the benefits of adaptation but also highlights that this ontological uncertainty in flood protection levels can strongly affect model outcomes. In addition, new research suggests that natural ecosystems should be incorporated as important means of protection against coastal (Arkema et al. 2013) and river (Stuerck et al. 2014) floods.

Validation of global and continental flood assessments is generally difficult as the scale is much larger than that of a single event. Given the rarity of flood events, establishing a meaningful compilation of relevant events with a full geographic spread would require hundreds of years of detailed observations of flood events, during which boundary conditions change (i.e. encroachment of floodplain, river training, weirs) (Lammersen et al. 2002). Theoretically, it is possible to derive observed estimates of global or continental flood risk using comprehensive databases of historical damaging events. This may not be available for all areas and would require the integration and harmonisation of databases from many different sources, for instance re-insurers and national governments. The global data bases NatCatSERVICE from Munich Re (www.munichre.com) and EM-DAT database^1^ are the most well-known databases that strive towards such a goal. An overview of event-specific databases for natural hazards on global, regional or national scale is provided by Tschoegl et al. (2006) and UNDP (2013).

### Macro (national) scale

#### Importance and use

Many countries have flood maps for their main cities and other vulnerable areas, particularly in Europe where this is stipulated by the European Union (EU) Floods Directive (2007/60/EC). This is much less common in developing countries though. In some countries, flood risk assessments have been performed at the national scale, meaning that a cartographic representation of flood information across the whole country has been developed, with a controlled degree of consistency in terms of accuracy and coverage. Such national scale assessments have a range of aims. In the USA, the national flood assessments have been performed to demarcate the limits of their national insurance programme (Burby 2001; Michel-Kerjan and Kunreuther 2011). In the UK, the national flood mapping programme has been designed to alert the public as to the risks that they face in their locality—by a web-based release of the maps—and to identify the total risk facing the country, in order to determine investment priorities and scale of government grant for flood risk management measures (Environment Agency 2009). In the Netherlands, decision-making on risk management strategies and assessing climate change impacts are the core reasons for the national flood risk mapping programme (such as *Waterveiligheid 21e Eeuw*; Kind 2011). In France, the identification of risk areas across the nation is fundamentally concerned with controlling development in such areas and also for investment prioritisation.

The different objectives of such national assessments generally lead to different scales within the national programme. For instance, national-scale climate change assessments are often quite broadbrush (Evans et al. 2004; Adaptation Sub-Committee 2012). Because in France, national maps are related to controlling local developments, a considerably greater level of detail is required—not least because decisions made based on that information are likely to be contested. The purpose of a national flood map does not only affect detail but also the indicators to be mapped. For project prioritisation, a number of return periods need to be represented on the map (Penning-Rowsell et al. 2013), whereas for spatial planning, just the flood extent of a design return period may be sufficient (DCLG 2010). This, then, raises the question what information the national flood maps should contain. Ideally, they should map extent (which is common) but also flood depths, velocities and similar information for future flood risks if climate change is exacerbating the hazard. The UK’s most recent national flood modelling is seeking to achieve this level of detail but this is likely to be expensive when applied nationwide.

#### Method

In terms of methodology, different approaches can be found. Sometimes, risk assessments at this scale are performed all at once, using a single model, applied nationwide (see Hall et al. (2003, 2005) and Evans et al. (2004) for the UK). In these cases, the consistency of the results between areas may be high, but the resources needed to model floods across a whole country are not to be underestimated. Moreover, given the large area, it is usually not possible to cover the entire territory with 2D hydraulic modelling and more simplified approaches are used, similarly to supra-national scale assessments. In order to estimate consequences, land use maps are used and combined with stage-damage curves, similar to meso-scale assessments. The resolution of such assessments is generally in the order of magnitude of 100- to1000-m grid cells. Another approach is to compile national flood maps bottom up as a composite of locally available information or as an aggregation of various meso-scale assessments (e.g. FLORIS in the Netherlands; RWS-DWW 2005). Whilst this increases the detail of the information and correspondingly enhances potential uses of a national-scale assessment, there is likely to be more inconsistency across the country.

#### Uncertainty and validation

Like supra-national flood assessments, verification or validation of the results is an issue with national assessments. No country is likely to have records of flood extent and other details of floods of long recurrence interval across the whole country. Therefore, some flood information is likely to be simulated and unverified and indeed unverifiable. One of the few examples from 10 or more years ago is for Hungary (Evans et al. 2000), where the country’s floodplains constitute 22.3 % of its area (compared to, for instance, 4.8 % in the UK) (Evans et al. 2000). The results of that study show that the potential 100-year event losses could be as high as 8 % of GDP or 35 % of the country’s annual public expenditure budget: very high figures and a threat to that country’s economy. Subsequent more detailed analysis of an intensively modelled Hungarian hot spot, showed that the national-scale results overestimated risk there by a factor of two (Evans et al. 2000).

Similarly, a detailed analysis of the UK national flood risk assessment, as conducted in 2008, also suggests that it exaggerates the flood risk facing the country by some four- or fivefold (Penning-Rowsell 2013, 2015). This may not matter in terms of prioritisation between different locations for investment, where the risk ranking may be the same irrespective of the absolute values, but it does lead to doubt as to the value of the risk assessment if the aggregate results are in error to this extent. A similar comparison in the USA found that more detailed modelling of flood risk areas, in comparison with a broad brush first estimate, showed inundated areas reducing in size markedly as more detailed information became available, which is problematic when one of the main aims of this mapping is to delimit the extent of risk for insurance purposes (Cook and Merwade 2009).

### Meso (regional) scale

#### Importance and use

There is also a strong need for sub-national flood risk assessments, for example at federal state level, for larger river stretches, large cities or on a basin-scale. Such meso-scale flood risk assessments are for instance used for regional flood risk management and mapping and in the re-insurance industry (e.g. Olsen et al. 1998; Ganoulis 2003). For example, in Germany, the federal states are responsible for flood management. Thus, many state authorities have undertaken flood hazard and risk assessments for their federal state to prioritise investments in structural flood mitigation measures and to provide hazard and risk maps for spatial planning purposes and public awareness raising (e.g. Sachsen, Bayern, Baden-Württemberg). Risk maps as presented in the Rhine-Atlas (ICPR 2001) are used for risk communication. Moreover, quantitative comparisons of different risks within a community or a region are undertaken on the basis of consistent risk assessments, such as for the city of Cologne, Germany (Grunthal et al. 2006).

Meso-scale studies are often used to evaluate the effect of certain management measures. Examples include the effect of retention areas on failure probabilities (Vorogushyn et al. 2012) and risk (De Kok and Grossmann 2010); restoration of abandoned meanders, a bypass, retention and reforestation (Te Linde et al. 2010b); spatial zoning and flood proofing (Poussin et al. 2012; De Moel et al. 2014a) and compartmentalisation and zoning (Koks et al. 2014a, b). Additionally, meso-scale assessments are often used to explore future scenarios, for instance, related to impacts of climate change (Gaslikova et al. 2011), socio-economic growth (Bubeck et al. 2011) or both (Te Linde et al. 2011; Elmer et al. 2012).

#### Method

The most common approach for meso-scale hazard assessment is based on the assumption of a spatially uniform return period. For example, a 100-year discharge is calculated for the entire river network as a basis to model inundation extent and depths (e.g. ICPR 2001; Bradbrook et al. 2005). The approach of spatially uniform return periods is very valuable if the assessment is used to answer local questions. However, it is misleading when assessing large-scale patterns, since it provides an unrealistic picture, overestimating meso-scale flood risk. This problem is avoided when a set of spatially consistent synthetic flood events with heterogeneous local return periods is generated (e.g. Rodda 2001, 2005; Falter et al. 2015b). Such stochastic flood event sets may be developed using multivariate statistical models that consider the spatial dependence between gauges (Lamb et al. 2010; Keef et al. 2013) or by starting with stochastic rainfall events (e.g. Rodda 2001). An alternative is a continuous modelling of rainfall-runoff, driven by continuous climate data or climate model scenarios (Falter et al. 2015b). An advantage of the latter is that hydrological processes influencing the runoff are implicitly considered and the complete flood event is consistently modelled for the whole catchment.

Physical processes like storage effects, flood attenuation or channel-floodplain interactions can be covered via complementing continuous rainfall-runoff modelling with a hydrodynamic simulation of inundation areas and water depth (Buchele et al. 2006, De Moel et al. 2009). Recent simplifications of fully hydrodynamic equations and reductions of computational model run time like parallelising enable 2D hydrodynamic simulations on larger scales. Such new approaches rely on coupled 1D/2D models where the channel flow is simulated one-dimensionally and the floodplain flows are simulated two-dimensionally with approaches ranging from simple-volume conservative storage-filling algorithms to fully dynamic shallow water modelling. Such meso-scale hazard assessments have been undertaken, for instance, for the Amazon (Wilson et al. 2007), the Ob (Biancamaria et al. 2009), the Pantanal (Da Paz et al. 2011) river basins and a 800-km reach of the Niger (Neal et al. 2012). However, due to computational constraints and data limitations, detailed hydrodynamic simulations are commonly avoided for meso-scale hazard assessments (e.g. ICPR 2001; Rodda 2005). Often simpler approaches are used based on rating curves and water surface intersection with topographic data (e.g. Ward et al. 2011).

Flood damage estimation on the meso-scale is commonly based on land use categories, which are connected to particular economic sectors. Exposure estimation needs to provide asset values also on the basis of land use units (e.g. Chen et al. 2004; Thieken et al. 2006; Wünsch et al. 2009). Losses are then estimated by aggregated sectoral stage-damage functions or multi-parameter models (Messner and Meyer 2006; Merz et al. 2010a, b; Kreibich et al. 2010; De Moel and Aerts 2011). Flood damage models are normally derived from micro-scale data related to single objects (Merz et al. 2010a, b). For instance, building-specific damage models are developed on the basis of empirical data collected in the aftermath of a flood (e.g. Merz et al. 2004) or on the basis of synthetic data collected by what-if analyses investigating which damage is expected in the case of certain flood situations (e.g. Penning-Rowsell et al. 2010, 2013, Tebodin 2000). Thus, scaling procedures for applications on the basis of land use units at the meso-scale need to be developed. For instance, Kreibich et al. (2010) developed an up-scaling procedure on the basis of land cover and geo-marketing data for flood loss estimation of companies in Germany. Tang et al. (1992) present an up-scaling procedure for residential, commercial, industrial and agricultural damage estimation based on average object areas and land use data for Bangkok, Thailand. In some cases, individual object information is used to derive flood damage estimates, but often this is done in an aggregated way. For instance, the HAZUS and HIS-SSM models both work with object counts (per census block and zip code, respectively) that are consequently considered as one unit in the damage calculation (Scawthorn et al. 2006; Aerts et al. 2014; Kok et al. 2005).

#### Uncertainty and validation

Uncertainties of meso-scale risk assessments have recently been the subject of various studies, addressing mainly aleatory and epistemic uncertainties (Apel et al. 2008, De Moel et al. 2012; 2014a, b; Freni et al. 2010; Merz and Thieken 2009; Saint-Geours et al. 2013). These studies illustrate the substantial uncertainty in absolute damage and risk estimates. Important contributors to this overall uncertainty relate to the probability of extreme events, the duration of such an event and the damage curves used to calculate the damage (see e.g. De Moel et al. 2014b). An important consideration at this scale for embanked regions concerns the possible failure of a levee (Vorogushyn et al. 2010), especially in areas with high safety standards and low elevations. Better validation of damage calculations is possible in cases where actual flood events and subsequent damage inventories have taken place (e.g. Seifert et al. 2010).

### Micro (local) scale

#### Importance and use

At the local—micro—scale, flood risk assessments are undertaken with detailed information about terrain elevation (e.g. via lidar data), hydraulic structures (e.g. dikes, weirs), building location/type/use, etc. Such local flood risk assessments are often undertaken to optimise investments via the evaluation of the cost-effectiveness of structural and other measures for flood risk reduction. Other purposes are the development of hazard and risk maps supporting the development of local flood management concepts and urban planning. Quantitative, spatially explicit risk information enables communities, companies and people to prepare for disasters (e.g. Takeuchi 2001; Merz and Thieken 2004). Examples of flood risk analyses at the city scale or even the sub-city scale include Cairns, Australia (Baddiley 2003); Gleisdorf, Austria (Neuhold and Nachtnebel 2008); Eilenburg, Germany (Apel et al. 2009); Dresden, Germany (Gerl et al. 2014; Kreibich et al. 2011a); New York City, USA (Aerts et al. 2013b; 2014), Ho Chi Min City, Vietnam (Lasage et al. 2014); Jakarta, Indonesia (Budiyono et al. 2015) and many more. Particularly, the distinction between meso- and micro-scale is slowly fading, with large cities being similar in size to certain regions and developments in computing power allowing highly detailed data to be used for larger areas.

#### Method

Hazard assessments are usually carried out using detailed 1D/2D hydraulic modelling (e.g. Ernst et al. 2010), for which a large variety of models exists. This can consider hydraulically important features like streets, buildings and channels (Aronica et al. 1998). Such models are based on solving physical equations for one/two dimensional flow of water, such as the Saint Venant equations (Aronica and Lanza 2005). Usually a flood frequency analysis is applied to a given record of discharge data of the relevant local gauge (e.g. Stedinger et al. 1993) to provide input for the hydraulic model and associate probabilities to the modelled inundation scenarios. Alternatively, rainfall-runoff models can be used to supply input hydrographs for the hydraulic model (as in Neuhold and Nachtnebel 2008). These hydraulic models do not only yield inundation extents and depths but can also provide additional flood indicators like flow velocity and the rate at which the water level rises. These can be important factors in determining the consequences of floods. For instance, flow velocity is an important parameter when assessing erosion and the collapse of buildings and the rate of rise is important when assessing potential fatalities. Modelling of the inundation area can be done using a full 2D model or by coupling 1D and 2D calculations in various degrees. For instance, the LISFLOOD-FP model links separate 1D channel flow calculations with 2D flood propagation calculations using uniform flow formulae (Bates and de Roo 2000).

In micro-scale assessments damage is often evaluated on an object level. For instance, in order to estimate the damage of a municipality, damages are calculated for each affected object, such as a building, business or school. Building-specific flood damage models are developed by collecting flood loss data in the aftermath of a flood or by undertaking synthetic analyses using experts, surveys or interviews (Merz et al. 2010a, b). On the basis of such empirical and synthetic data, generalised relationships between damage and influencing parameters such as flood characteristics and resistance factors are derived (e.g. Green 2003; Penning-Rowsell et al. 2010, 2013; Kreibich et al. 2010). Studies have shown that estimations based on stage-damage functions may be very uncertain since water depth and building use only explain a part of the data variance (Merz et al. 2004; Schröter et al. 2014). Whilst conceptually the same as meso-scale assessments using depth-damage curves, micro-scale assessments have the potential to better differentiate generic classes (e.g. between residential or industrial buildings) as there is less heterogeneity in such a class at the micro-scale as opposed to the meso-scale (i.e. less variation in types of houses). Moreover, at the micro-scale, there is the possibility for site visits and individual inspection to specify or improve damage curves. Neuhold and Nachtnebel (2008), for instance, undertook surveys and interviews with chief operating officers for their case study in Gleisdorf. In their experience, the interviews helped improve the quality of the data underlying depth-damage curves remarkably. However, uncertainty remains relatively high, so that the development of probabilistic damage models seem to be an innovative enhancement, since they inherently provide quantitative information about the model uncertainty (Vogel et al. 2013; Merz et al. 2013; Schröter et al. 2014).

#### Uncertainties and validation

At the micro-scale, epistemic uncertainties with respect to hydraulic modelling become apparent. These are usually associated with the selection of the appropriate model parameterization, the consideration of dikes and dike breaches and the calibration and validation of the models (Apel et al. 2009). Validation of inundation extents is quite common, for instance, against RS imagery of observed floods (Schumann et al. 2009; Schumann and Di Baldassarre 2010). Less common, though more common than at larger scales, is the validation of modelled damages at the micro-scale, as observational data are still quite rare (Merz et al. 2010a, b). A noticeable exception is Thieken et al. (2008), who validated FLEMOps for five municipalities that were affected by the German floods in 1993 and 2002 and also compared their results to those found using stage-damage curves from some other models. Similarly, Jongman et al. (2012b) employed seven damage models on two cases (Carlisle and Eilenburg) and compared them to observed damages for these cases. Specifically for the commercial sector, Seifert et al. (2010) validated FLEMOcs. These results show that there is a considerable variation in results between damage models and the absolute estimates often compare poorly to the observed damages.

## Discussion and comparison across scales

The preceding sections illustrate that there are considerable differences in the methodology and purpose of flood risk assessments at different scales (Table 1). Methodology and purpose are very much linked. Large-scale (supra-national, macro-) assessments are less detailed in terms of lower spatial resolution and generate more generalised information on potential consequences. However, this does allow for a methodologically consistent assessment over the entire area of interest, for instance in the case of national insurance or international re-insurance purposes. Table 1 gives an overview of the findings of the preceding section on several characteristics at different scales.

**Table 1:** Overview of characteristics of assessments at different scales

	Supra-national (global/continental)	Macro (national)	Meso (regional, province)	Micro (local, city)
Resolution (DEM)	1–10 km	100 m–1 km	∼25–100 m	∼1–25 m
Hazard estimation	-Global river flood model -Surge heights (coastal)	-Generic flood model -Aggregation of hydraulic simulations	-Rainfal-runoff plus 2D hydraulic modelling or simplification	-2D hydraulic modelling
Consequence estimation	-Gridded GDP or population in flood zone	-Land use and stage-damage curves	-Land use and stage-damage curves	-Stage-damage curves for individual buildings
Uncertainty	-Presence of flood defences important unknown (ontological)	-Inundation modelling at course scale (ontological/epistemic)	-Failure of defences (epistemic) -Probability event (aleatory) -Damage calculation (epistemic)	-Hydraulic modelling (epistemic) -Failure of defences (epistemic) -Probability event (aleatory) -Damage calculation (epistemic)
Validation	Aggregated datasets (EM-DAT, NatCatSERVICE)	Absolute totals seem overestimated	Limited to aggregate damages	Possible for recent events with well-documented damages
Academic application	-Assessment -Effect climate change and population growth	-Assessment -Effect climate change and population growth	-Effect of measures -Future developments -Uncertainty analyses	-Effect of measures -Evaluate different strategies -Validation of methodologies
Societal use	-Disaster relief funds -Re-insurance -Multi-nationals	-National insurance programme -Communication and awareness -Controlling local development	-Prioritisation investments -Support planning -Communication and awareness	-Evaluating specific measures -Optimise investments -Support local management and planning

Any assessment is usually limited to modelling a single flooding process, such as flooding of a river valley by rising water levels (valley flooding) or the flooding of a large polder area by water propagating through it from a single source (such as a dike breach). At the micro- or meso-scale, this is much less often an issue, as the area under investigation is likely more homogeneous. In larger-scale assessments, this can lead to erroneous results for regions where another type of flooding process is dominant. For instance, in pan-European studies on river flooding (e.g. Alfieri et al. 2013), results for the Netherlands usually do not seem very plausible as it is dominated by large polder areas.

### Flood protection works

The above issue is also linked to the presence of flood protection works, which seems to be a concern at all scales. At a large scale, protection works are usually not included at all, though there are some approaches where this is corrected by taking the integral up to a defined protection level in the risk calculation (e.g. Feyen et al. 2012; Ward et al. 2013). A database on actual protection levels is however often lacking, though Hallegatte et al. (2013) made a first attempt for coastal cities and Jongman et al. (2014) developed a model to estimate protection levels in Europe based on potential damage without protection. At a smaller scale, there is often a considerable uncertainty on where or when such a defence may fail. At small scales, this can be approached using the so-called fragility curves, which denote the probability of failure given certain hydraulic conditions (e.g. Vorogushyn et al. 2009). It remains uncertain, however, to estimate failure conditions of objects for situations that they have never faced, especially given the long length of levees and heterogeneity of their composition and subsoil.

#### Uncertainty

Uncertainty plays a role in assessments at all scales. Besides the way protection works are addressed, another element that is common across scales relates to uncertainty in the probability of hazard events. There is substantial aleatory uncertainty in the (relatively short) time series of annual maxima, on which frequency distributions are based that denote the probability of an event with a specific magnitude. Model uncertainty is usually an epistemic source, for instance, the type of distribution used to characterise the frequency has a considerable influence (Xu et al. 2007). This is especially relevant for the assessment of extreme events on the basis of short time series, causing large uncertainties in the extrapolation range (Apel et al. 2008). Further problems might be the violation of the underlying assumptions of stationarity and homogeneity of the time series. Epistemic uncertainty associated with flood damage estimates results from uncertainty in the estimation of exposed assets (e.g. due to bias in spatial disaggregation, uncertainty of asset estimates derived from regional statistics) and from uncertainty in the depth-damage functions (e.g. due to transfer of damage functions derived from other regions and other flood events, disregard of many damage influencing factors, such as flood experience, flood duration or flow velocity) (Merz and Thieken 2009). Most comparative uncertainty analyses have been performed at the micro- and meso-scales, identifying key elements that affect risk assessments (e.g. Apel et al. 2008; Merz and Thieken 2009; De Moel et al. 2012, 2014b). The most apparent ontological uncertainty relates to large scales and involves the presence of protection works. At smaller scales various sources of epistemic uncertainty are important. However, it should be remembered that also at smaller scales assessments can easily miss out on important aspects of flood risk, for instance, related to specific sectors that may not be included (such as infrastructure), indirect economic effects or damage-influencing factors such as experience and the preparedness of the population.

#### Validation

Another issue that is relevant at all spatial scales concerns the validation of results. Validation of flood risk assessments is sparse, at all scales, mainly due to data limitations. However, the need for validation is evident as uncertainties in flood risk estimates are generally found to be quite large (Apel et al. 2008; De Moel et al. 2012; 2014a, b; Seifert et al. 2010; Penning-Rowsell 2013, 2015). Moreover, studies using several damage models on a single case that include comparisons to observed damage (e.g. Apel et al. 2009; Jongman et al. 2012b) show substantial discrepancies and a large range between models. If validation of flood risk estimates is sparse, the credibility of such estimates may be harmed. A case in point is the apparent overestimation of risk at larger scales (Penning-Rowsell 2013, 2015). Bouwer et al. (2009) even found that using a 100-m grid instead of a 25-m grid for the same case study area already resulted in damage estimates up to 50 % higher, mainly because the relative share of urban land use classes increases at a lower resolution. These studies thus suggest that assessments using a higher level of detail result in lower estimates of risk. There can be various reasons for this, such as the use of the full value at risk as damage, overestimating the fraction of urban land use or using spatially uniform return periods over a large area (larger than a single event).

#### Vulnerability

The consequences of flooding mainly relate to physical assets, with vulnerability used in the context of how easily assets get damaged by floods (i.e. the depth-damage curves). However, the concept of vulnerability can have a much broader meaning in the context of flooding. This may also relate to the vulnerability of people (e.g. ill or elderly people) or the recovery capacity of households, businesses and society. Such considerations are, however, very rarely included in flood risk assessments at any scale. Anecdotal evidence from studies at local to regional scales suggests that societies become less vulnerable over time. Also, several studies show that people and societies may learn from past disasters (e.g. Burby and French 1981; Wind et al. 1999; Cutter et al. 2008; Kreibich and Thieken 2009; Yamamura 2010; Kreibich et al. 2011b), and some studies (Hallegatte 2007; Crompton and McAneney 2008) show that damages may decrease following a disaster because of behavioural changes and adaptation (Bubeck et al. 2012). Yet, such temporal dynamics of vulnerability’s influence on risk are rarely studied (except for Peduzzi et al. (2012) for tropical cyclone risk). Vulnerability can also relate to the functioning of the system instead of the behaviour of individuals. In this case, the functioning of critical infrastructure is a key, but has rarely been included in flood risk assessments, usually resulting from a lack of knowledge and data of the system. As it has a high importance for local policy makers, many activities are starting to investigate this at the local scale, for instance, in the wake of Hurricane Sandy (Tsay et al. 2014).

#### Linking spatial scales

Various issues play a role at all scales and thus warrant attention in future research. However, there are also opportunities opening up for up- and downscaling between spatial scales. This is driven by increased data availability and increased computing capacity. Where macro-scale assessments are usually based on land use categories, there are now various countries with datasets of individual buildings. Correspondingly, damage and risk methodologies, formerly mainly used at the micro-scale, can in some regions be scaled up to the meso- and macro-scales. Building extraction techniques from remote sensing (Ehrlich and Zeug 2010; Freire et al. 2010; Gerl et al. 2014) can further push this development by providing building footprints across the globe. In the other direction, global flood hazard assessments are becoming more and more detailed, allowing for opportunities to downscale. When such global flood hazard assessments can be linked to meso-/micro-scale damage calculations, it will become possible to perform studies to support flood risk decision-making in areas that lack detailed hazard information. This may need further detail in global hazard assessments or a smart way to efficiently downscale global hazard information to specific areas. When combined with emerging global high-resolution datasets on exposure (such as the Global Human Settlement Layer (GHSL) from JRC), flood protection (Hallegatte et al. 2013; Jongman et al. 2014) and damage curves (Huizinga 2007), this also allows for consistent assessment across the globe to support global actors such as the Red Cross, World Bank and re-insurers.

## Conclusions

This review addressed methodologies and uses of flood risk assessments at various spatial scales. It shows that spatial scale, method and use of assessments are closely linked. At large scales (supra-national, macro) geographically consistent assessments of flood risk aid the international (re-) insurance industry or can be used to identify risk hotspots by international financing institutes in order to prioritise investments. At smaller scales (meso, micro), assessments can be tailored to a specific basin, city or site in order to directly support decision-making in that area by evaluating the (cost-) effectiveness of different types of measures.

Whilst there are clear differences between scales, boundaries are slowly fading due to increased computing power and availability of higher detailed harmonised datasets. In this regard, the links between assessments at different scales is also emerging as a promising subject. For instance, a well-developed link between the global and meso-/micro-scale would enable rapid assessments of flood risk for local policy makers in regions where few data are available. The use of global datasets for information on the hazard, exposure and vulnerability would also allow for more consistent comparison of flood risk in cities across the globe.

This review showed that important developments have taken place in recent years in developing global flood risk assessments. However, also at other spatial scales, many developments are taking place, for instance, at the local level which benefits greatly from the increased availability of high-resolution data for the determination of the hazard and consequences (LiDAR data, databases of buildings and uses). Through this review, important topics for further development have been identified. At the supra-national scale (and to a lesser degree macro-scale), there is a clear need for the inclusion of flood defences in assessments, for which the development of a database on flood defence structures and protection levels is necessary, as well as methodologies to incorporate these data. At the macro- and meso-scale there are opportunities to broaden assessment from direct effects to also include indirect economic effects. For this, better differentiation of the impact of flooding on different sectors is necessary in order to be able to directly link results of flood assessments to economic (input-output or general equilibrium) models. At the meso-/micro-scale, the topic of critical infrastructure (i.e. electricity, gas, water, telecoms networks) is emerging as a key caveat, which has a high relevance for policy makers. Insights from other disciplines, for instance, related to terrorism (Patterson and Apostolakis 2007) or volcanic (Wilson et al. 2012) risks, and network models in general (e.g. Dueñas-Osorio et al. 2007; Winkler et al. 2011) could also prove to be very useful for flood risk management.

Lastly, validation of flood risk models is an issue at all scales. Flood risk assessment in absolute numbers are surrounded by considerable uncertainties, and some studies have shown that validation in real-life cases gives mixed results (e.g. Jongman et al. 2012b). At the same time, encouragingly good results have been reported (e.g. Aerts et al. 2014). More event-based work (as opposed to assuming a uniform return period over a large area) would allow for better validation opportunities, but would also require increased efforts in gathering post-disaster information on the consequences of actual floods. This would, at the same time, help to improve the risk models themselves, for instance, because of better differentiation of depth-damage curves and the identification of important contributing categories, such as infrastructure damage, the costs of emergency management and temporal relocation.

## Footnotes

1
http://www.emdat.be/
